# A new mode of action for unconventional NMDA receptors

**DOI:** 10.7554/eLife.107049

**Published:** 2025-05-19

**Authors:** Ikuko Smith

**Affiliations:** 1 Department of Molecular, Cellular and Developmental Biology, Department of Psychological and Brain Sciences, University of California, Santa Barbara Santa Barbara United States

**Keywords:** hebbian learning, synaptic plasticity, long-term depression, presynapses, postsynapses, NMDA receptor, Mouse

## Abstract

Presynaptic NMDA receptors can shape timing-dependent long-term depression in a way that departs from their classic postsynaptic role.

**Related research article** Thomazeau A, Rannio S, Brock JA, Wong HH, Sjöström PJ. 2025. Neocortical Layer-5 tLTD Relies on Non-Ionotropic Presynaptic NMDA Receptor Signaling. *eLife* **14**:RP106284. doi: 10.7554/eLife.106284.

The mnemonic “Cells that fire together, wire together” encapsulates a principle known as Hebbian learning. Named after the highly influential McGill University psychologist Donald Hebb, this theory suggests that synapses, the connections between neurons, can be modified through experience – a process referred to as synaptic plasticity (Hebb, 1949).

Hebbian learning relies on precise timing between the activation of two connected ‘presynaptic’ and ‘postsynaptic’ neurons (Bi and Poo, 1998). For effective synaptic modification to take place, both cells need to ‘fire’ (emit action potentials) within tens of milliseconds of each other. If the presynaptic neuron is activated first (causal timing), the synapse is strengthened, leading to long-term potentiation, or LTP (Bliss and Lomo, 1973). If the postsynaptic neuron spikes before the presynaptic neuron (acausal timing), the synapse is weakened, resulting in long-term depression, or LTD (Dudek and Bear, 1992). But how do these neurons determine which one spiked first?

At the core of timing-dependent plasticity is the N-methyl-D-aspartate (NMDA) receptor, one of the three classes of neurotransmitter receptors located on the postsynaptic side of excitatory glutamatergic synapses. These receptors are ionotropic – that is, they allow calcium and sodium ions to enter the cell upon glutamate binding.

One additional quirk of this receptor is that its pore is blocked by magnesium ions when the neuron is at rest. To allow ions to flow through the channel, a positive increase in the membrane potential (depolarization) is necessary to repel the magnesium ions from the pore (Burnashev et al., 1992). Thus, the NMDA receptor needs two simultaneous events to be activated: for the presynaptic neuron to have just fired and therefore to have released glutamate, and for the postsynaptic neuron to be depolarized enough to remove the magnesium ion blocking the pore. This requirement makes the NMDA receptor an ideal coincidence detector that is maximally activated when the presynaptic and postsynaptic neurons fire together (Seeburg et al., 1995).

When NMDA receptors are properly activated, the resultant influx of calcium into the postsynaptic neuron serves as a central mediator of synaptic plasticity by triggering a complex cascade of cellular events that ultimately strengthen the synapse. If the postsynaptic neuron spikes at an acausal timing, however, the influx of calcium ions through the NMDA receptor is reduced and the synapse weakened (Graupner and Brunel, 2012). Thus, the ionotropic role of NMDA receptors has long been strongly linked to the cellular mechanisms for plasticity.

Although the properties of the NMDA receptor align well with the principles of the Hebbian rule, several issues arise. First, NMDA receptors have also been found on the presynaptic side where they cannot act as a coincidence detector (DeBiasi et al., 1996). Second, there is evidence of unconventional non-ionotropic NMDA receptor signaling that seems to take place without ions flowing through the channel (Dore et al., 2015). How these intriguing presynaptic NMDA receptors contribute to synaptic plasticity is not fully understood, especially as they are not consistently observed at synapses, unlike ionotropic receptors (Christie and Jahr, 2009; Duguid, 2013).

Now, in eLife, Aurore Thomazeau, Sabine Rannio, Jennifer A Brock, Hovy Ho-Wai Wong and P Jesper Sjöström from McGill University, Université Côte d’Azur, and the Chinese University of Hong Kong report that timing-dependent LTD (tLTD) in certain neurons depends on presynaptic, non-ionotropic NMDA receptors, but not on postsynaptic ones (Thomazeau et al., 2025; Figure 1). These conclusions were reached using a multitude of technical approaches in mouse neurons from the visual cortex.

**Figure 1.**
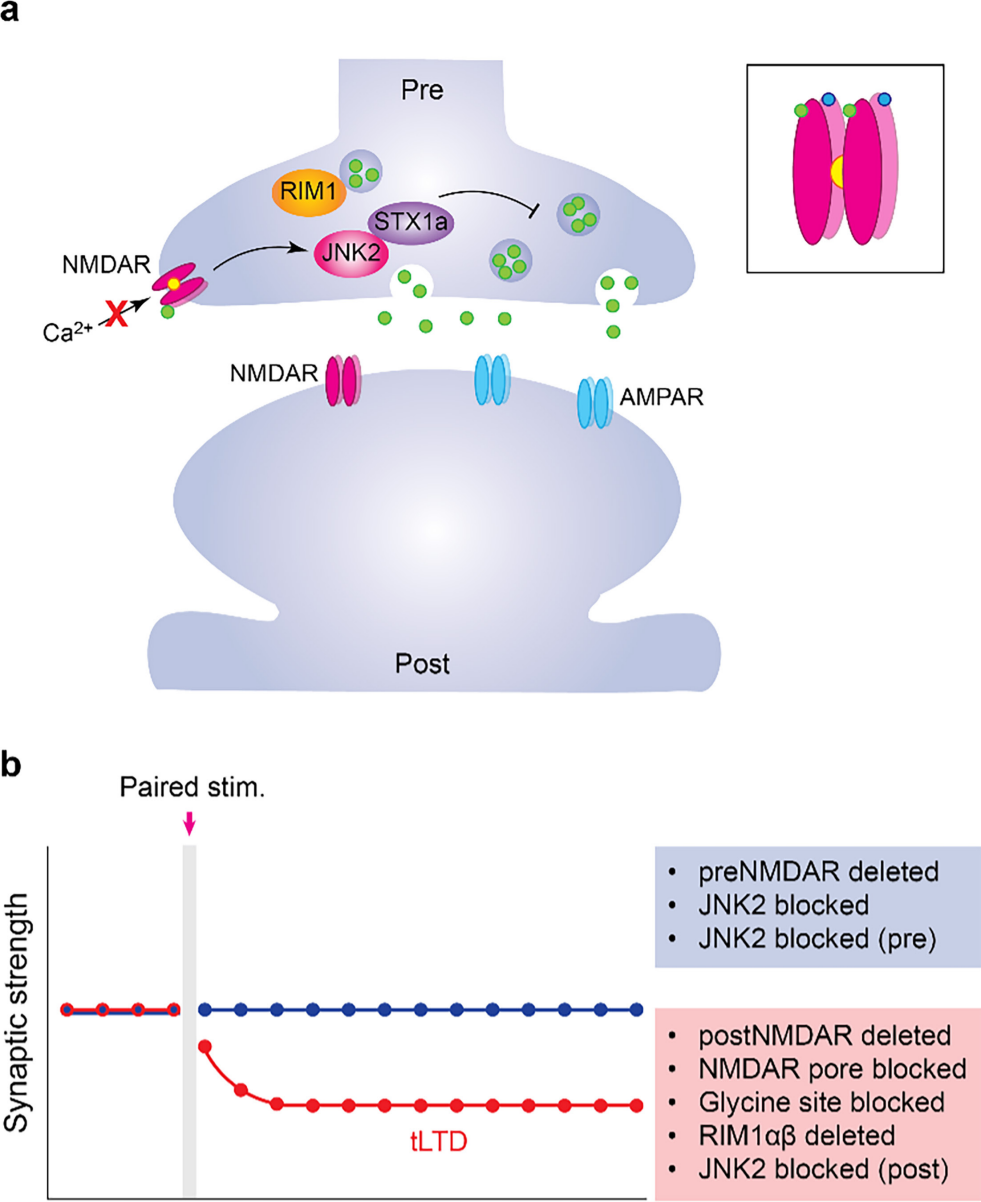
Timing-dependent, long-term synaptic depression (tLTD) Timing-dependent, long-term synaptic depression (tLTD) in Layer 5 visual cortical neurons relies on presynaptic NMDA receptor signaling that does not require the influx of calcium ions through the channel. (**A**) Certain unconventional NMDA receptors (NMDAR; pink) are present on presynaptic membranes (top), even though they are most known for their roles at the postsynaptic membrane (bottom). NMDA receptors are typically activated when two molecules each of glutamate (green) and the cofactor glycine (blue) bind to them, but the channel remains blocked by magnesium (Mg2+; yellow) in the absence of membrane depolarization (inset). This prevents calcium ions from entering the cell (crossed arrow) and triggering further cellular events. Another class of glutamate receptor (AMPAR; blue) is also involved in synaptic transmission. Thomazeau et al. shed light on the presynaptic NMDA receptor-dependent mechanism for tLTD. They revealed that it involves JNK2 signaling, which is known to regulate neurotransmitter release. JNK2 signaling is mediated by close interaction with its partner, Syntaxin-1a (STX1a). Genetically deleting another presynaptic molecule, RIM1αβ, on the other hand, did not affect tLTD. (**B**) Disruption of presynaptic NMDA receptor or JNK2 signaling in the presynapses abolished tLTD (static blue line), and synaptic connections were not weakened. However, deleting NMDA receptors from postsynaptic neurons left tLTD induction intact (red line), weakening the synaptic connection.

Deleting NMDA receptors from presynaptic neurons disrupted tLTD but deleting them from postsynaptic neurons did not affect the plasticity (Figure 1B). Additionally, pharmacological treatments that preserved glutamate binding but blocked the pore of the receptors or prevented the channel from opening did not impact tLTD. Collectively, these results suggest that tLTD in these neurons depends on a non-ionotropic, presynaptic NMDA receptor function that does not involve calcium influx through the channel.

To further investigate this mechanism, Thomazeau et al. focused on two presynaptic molecules, RIM1αβ and JNK2, which are known to affect glutamate release in a presynaptic, NMDA receptor-dependent mode (Abrahamsson et al., 2017). Genetically deleting RIM1αβ did not affect tLTD, while pharmacologically blocking JNK2 signaling abolished it. Furthermore, blocking JNK2 signaling in the presynaptic neuron, but not in the postsynaptic one, affected the expression of tLTD. These findings confirmed the presynaptic origin of tLTD induction and identified JNK2 as a key partner of the presynaptic NMDA receptor, which regulates glutamate release and causes synaptic depression.

The challenging yet elegant experiments revealed the role of presynaptic NMDA receptors in a specific form of plasticity found in a deep layer of the visual cortex. A compelling theorem can sometimes lead us astray. Although the existence of presynaptic NMDA receptors has been known for quite some time, their functional role in synaptic plasticity had gone unnoticed, as we expected them to act as ionic channels fit for coincident detection. The unconventional presynaptic NMDA receptor-dependent form of synaptic plasticity adds to the growing list of recently discovered types of plasticity, such as behavioral time scale synaptic plasticity and non-Hebbian heterosynaptic plasticity (Bittner et al., 2017; Faress et al., 2024). The diversity of these plasticity rules allows our brains to be adaptable. It remains to be seen whether and how different neurons and synapses adopt varying plasticity rules.
